# The effect of additional protein on lean body mass preservation in post-bariatric surgery patients: a systematic review

**DOI:** 10.1186/s12937-021-00688-3

**Published:** 2021-03-14

**Authors:** Marleen M. Romeijn, Daniëlle D. B. Holthuijsen, Aniek M. Kolen, Loes Janssen, Goof Schep, François M. H. van Dielen, Wouter K. G. Leclercq

**Affiliations:** 1 Department of Surgery, Máxima Medical Center, De Run 4600, Veldhoven, 5504 DB The Netherlands; 2 Research School NUTRIM, Department of Surgery, Maastricht University Medical Center, Maastricht, the Netherlands; 3 Maastricht University, Faculty of Health, Medicine and Life Sciences, Maastricht, The Netherlands; 4 Department of Sport Medicine, Máxima Medical Center, Veldhoven, The Netherlands

**Keywords:** Bariatric surgery, Protein intake, Body composition, Lean body mass, Systematic review

## Abstract

**Background:**

As result of bariatric surgery, patients are susceptible to protein deficiency which can result in undesirable lean body mass (LBM) loss. Consumption of high-protein diets or supplements could counteract this, but evidence about the effect is scarce. This paper systematically reviewed the literature to determine the effect of additional protein intake (≥60 g/day) on LBM preservation in post-bariatric patients.

**Methods:**

An electronic search of PubMed, EMBASE and the Cochrane Library was conducted. Studies were included if patients received a high-protein diet or protein supplements for at least one month, and LBM was assessed. The primary outcome was difference in mean LBM loss between the experimental (protein) and control group. Secondary outcomes were differences in body fat mass, total body water, body mass index and resting metabolic rate.

**Results:**

Two of the five included studies (*n* = 223) showed that consumption of proteins resulted in significant LBM preservation. Only one study reported a significant difference in the reduction of body fat mass and resting metabolic rate in favour of a high-protein diet, but none of the studies showed a significant difference in total body water loss or body mass index change between the two groups.

**Conclusions:**

This paper showed inconclusive evidence for LBM preservation due to protein supplementation or a high-protein diet in post-bariatric patients. This outcome might be subjected to certain limitations, including a lack of blinding and a low compliance rate reported in the included studies. More specific and personalized recommendations regarding protein intake may need to be established by high quality research. Studies investigating the quantity (g/day) and quality (whey, casein or soy) of proteins are also needed.

**Supplementary Information:**

The online version contains supplementary material available at 10.1186/s12937-021-00688-3.

## Introduction

Bariatric surgery (BS) is considered the most effective treatment for severe obesity [1–3]. Despite the successful weight loss, patients are prone to develop nutrient deficiencies due to energy restriction, malabsorption and food intolerances [4, 5]. Current guidelines recommend patients to consume 60-80 g proteins a day or 1.2 g/kg of the ideal body weight (IBW) [6–8], but adherence to these guidelines is known to be problematic in 45% of BS patients [4]. There is a substantial prevalence of excessive lean body mass (LBM) loss in BS patients. Within the first year after laparoscopic Roux-en-Y gastric bypass (RYGB), patients lose about 22% of their LBM [9, 10]. LBM plays an important role in resting energy expenditure, functional capacity, muscle strength and cardiovascular health [11–13]. In post-bariatric surgery patients, an excessive loss of LBM can be detrimental as it may slow down weight loss or even trigger weight regain [14–16]. Mechanisms behind this are a reduced resting metabolic rate (RMR) and a direct change in appetite [14, 16–18]. Moreover, an inadequate protein intake (≤60 g/day) potentially results in decreased feelings of satiation and decreased diet-induced thermogenesis, which may hinder weight loss [19]. For these reasons, an adequate protein intake and preservation of LBM in BS patients is of significant importance in long-term weight management.

There is a paucity of data that shows the correlation between protein intake and LBM loss after BS. In 2017, a systematic review concluded that two of the four studies with an adequate protein intake (≥60 g/day) was associated with significantly less LBM loss one year after RYGB [17–19]. A major criticism of this review is that the protein intake in eight studies was relatively inadequate (< 60 g/day) generating insufficient evidence. Currently, no systematic review has investigated the effect of an adequate protein intake (≥60 g/day) achieved by high-protein diets or protein supplements on LBM preservation, while multiple randomized controlled trials (RCTs) have been performed. Therefore, the aim of this systematic review was to evaluate the effect of protein supplementation or a high-protein diet (≥1 month) on LBM preservation in post-bariatric surgery patients, compared to patients following standard treatment.

## Method

This review complies with the recommendations of the Cochrane Handbook for Systematic Reviews and Interventions [20] and was recorded according to the PRISMA systematic review guidelines [21]. The systematic review protocol was registered in the International Prospective Register of Systematic Reviews (PROSPERO) under registration number CRD42020176839.

### Search strategy

The systematic search was performed in February 2020 and was conducted in three electronic databases: MEDLINE (PubMed Legacy), EMBASE (Ovid) and The Cochrane Library. The search included only human studies that were published in English or Dutch, and was not restricted by publication date. Keywords in the search strategy included [dietary protein], [protein supplementation] and [bariatric surgery], and their synonyms. The full search strategies for all databases can be found in supplementary Table 1. References of relevant reviews and included studies were hand searched for potential eligible studies that have been missed.

### Eligibility criteria

Studies were considered eligible if they included: 1) patients in the age of 18–65 years with a body mass index (BMI) of ≥35 kg/m^2^ who underwent RYGB or sleeve gastrectomy (SG), 2) daily protein supplementation or a high-protein diet for ≥1 month (≥60 g/day), started within 2 weeks after surgery, compared to standard treatment (control), 3) body composition as outcome measurement determined by either air displacement plethysmography, bioelectrical impedance analysis (BIA), dual-energy X-ray absorptiometry (DXA) or magnetic resonance imaging (MRI), 4) a follow-up of ≥2 months, and 5) an experimental or observational study design including a control group. Exclusion criteria were 1) inclusion of pregnant women, 2) protein supplementation or a high-protein diet combined with ≥2 times supervised strength training per week, without data about the effect of proteins only, 3) no data about primary outcome (LBM), or 4) reviews, letters, case series, case reports, conference abstracts and editorials.

### Study selection

Initial records were screened for relevance on titles and abstract. Full-texts of relevant articles were obtained for checking final inclusion. Endnote X9 software was used to manage all references, including removal of duplicates.

### Data extraction

The following data was extracted by one researcher (DH) using a standardized study form: authors’ names, publication year, study design, follow-up period, sample size, gender, mean age, mean BMI, baseline LBM, surgery type, intervention protocol, protein intake prior to surgery, actual protein intake, compliance and study outcomes (LBM, body fat mass (BFM), total body water (TBW), BMI and RMR. A second author (MR) cross-checked the information.

### Study outcomes

The primary outcome was difference in mean LBM loss between the experimental (protein) and control group. Secondary outcomes were differences in BFM, TBW, BMI and RMR. If no score (in kg or %) of the predefined outcome was provided, a score was calculated based on the available data (pre- and post-surgery). Effect sizes of the individual studies were calculated using Cohen’s d. An effect size of ≤0.2 was considered trivial, 0.2–0.49 was considered small, 0.5–0.79 was considered moderate and ≥ 0.8 was considered high [22].

### Quality assessment

Study quality was assessed by the Cochrane Collaboration’s Risk of Bias Tool [23]. The Cochrane Collaboration’s Risk of Bias tool subdivides studies into “low”, “unclear” or “high” risk for various biases (selection bias, performance bias, detection bias, attrition bias, reporting bias and other bias). Two reviewers (DH, MR) judged the quality of each individual study based on a set criteria. Any disagreements were solved by a third reviewer (LJ).

## Results

### Study selection

**Fig. 1:**
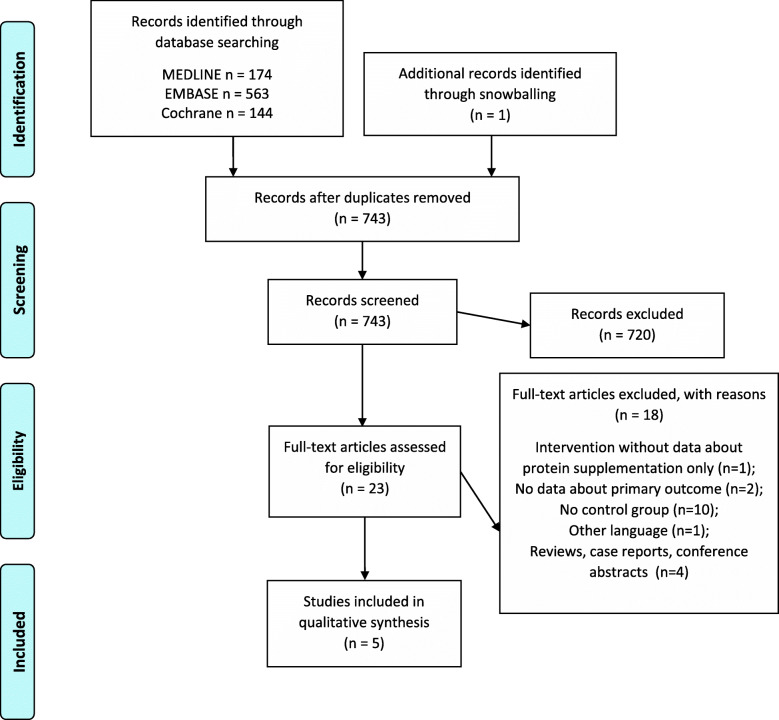
PRISMA flow diagram showing selection of articles

In total, 881 articles were identified in three electronic databases and one article was identified in a reference list. After duplicate removal, 743 articles remained. After screening of titles and abstracts, 23 potentially relevant articles were selected for full-text reading. At the end, five studies met the inclusion criteria and were considered eligible for this systematic review [24–28] (Fig. 1).

### Study characteristics

The sample sizes of the included studies varied from 20 [28] to 60 [25, 27] patients (Table 1). The follow-up periods ranged from 8 weeks [24] to 6 months [25, 26, 28] and 12 months [27]. Two studies included only SG patients [25, 27], two studies included only RYGB patients [24, 26] and one study included both types of BS [28]. Three of the five studies used protein supplements [25, 26, 28], one study used amino acid supplements [24] and one study used a protein-enriched diet to increase daily protein intake [27]. The dose of protein supplements or protein content in high-protein diets varied from 15 g/day [28] to 2.0 g/kg IBW/day [27]. Two of the included studies reported a high level of patients’ compliance [27, 28], whereas two studies reported a low level [25, 26] and one study did not asses compliance [24]. Some authors reported reasoning for the low compliance, proposing that this can be improved by closer follow-up and face-to-face interviews [25]. Three of the five studies assessed body composition by BIA [25, 27, 28], whereas the other two studies assessed body composition using DXA [24, 26].

**Table 1:** Study characteristics of the included studies

Author, year	Study design, follow-up	Sample size Gender	Age (yr) BMI (kg/m^**2**^) LBM (kg)		Surgery type	Intervention	Pre-surgery protein intake (g/day)	Actual protein intake (g/day)	Compliance	Protein intake analyzed	Outcomes analyzed
			CON	PRO							
Clements et al., 2011 [24]	Unblinded randomized control pilot study, 8 weeks	30 M (n = 1) F (*n* = 29)	46.0 ± 7.5 43.6 ± 4.2 54.0 ± 8.1	47.9 ± 9.6 42.9 ± 4.1 52.4 ± 6.9	RYGB (*n* = 30)	CON: usual care, no use of protein supplements PRO: oral supplement containing 24 g leucine metabolite, glutamine and arginine twice daily for 8 weeks	*NA*	*NA*	*NA*	Log sheets	TBW, BMI Body composition using DXA (BFM, LBM) RMR
Günes et al., 2019 [25]	Randomized controlled trial, 6 months	60 M (*n* = 9) F (*n* = 51)	43.5 ± 8.4 45.9 ± 6.5 61.7 ± 13.3	40.3 ± 11.4 46.2 ± 5.0 47.5 ± 13.5	SG (*n* = 60)	CON: standard diet for 1 month, no use of protein supplements PRO: standard diet + 1,2 g/kg IBW/day protein (±80 g) whey powder for 1 month	*NA*	CON: *NA* PRO: 51	38%^a^	*NA*	TBW, BMI Body composition using BIA (BFM, LBM)
Oppert et al., 2018 [26]	Randomized controlled trial, 6 months	53 F (*n* = 53)	43.9 ± 10,7 43.6 ± 6.2 55.6 ± 8.4	42.5 ± 8.7 43.3 ± 6.0 55.9 ± 6.1	RYGB (n = 53)	CON: usual care with general dietary and physical activity counselling PRO: oral supplement containing whey-protein-enriched powder (24 g) twice daily for 6 months	CON: 80 PRO: 82	CON: 60 PRO: 82	55%	Dietary history method. Return of empty cans of protein powder	TBW, BMI Body composition using DXA (BFM, LBM)
Schiavo et al., 2017 [27]	Randomized comparative study, 12 months	60 M (*n* = 60)	41.0 ± 6.2 40.7 ± 5.3 75.0 ± 11.9	43.0 ± 5.5 42.1 ± 6.2 76.3 ± 7.0	SG (n = 60)	NPD (normal protein diet): 1.0 g/kg IBW/day protein (23.3%), 15% fat and 61.7% carbohydrates for 12 months PED (protein-enriched diet): 2.0 g/kg ideal IBW/day protein (±160 g)(47.7%), 15% fat and 37.7% carbohydrates for 12 months	*NA*	CON: 67 PRO: 143	Very high	Questionnaires, 3 day dietary record, 72 h-recall	TBW, BMI Body composition using BIA (BFM, LBM) RMR
Schollenberger et al., 2016 [28]	Randomized controlled double-blind pilot study, 6 months	20 M (n = 3) F (*n* = 17)	47.0 ± 11.9 49.0 ± 5.1 68.9 ± 13.4	43.4 ± 13.3 52.0 ± 7.6 65.2 ± 14.2	RYGB (n = 5) SG (*n* = 15)	CON: pure maltodextrin powder, 15 g/day first month, 30–35 g/day second-sixth month PRO: milk protein powder, 15 g/day first month, 30–35 g/day second-sixth month	CON: 93 PRO: 97	CON: 53 PRO: 67	86%	4 day dietary record, interviews	TBW, BMI Body composition using BIA (BFM, LBM)

Values are expressed as mean ± SD

List of abbreviations: *CON* control group, *PRO* protein group, *RGYB* Roux-en-Y gastric bypass, *SG* sleeve gastrectomy, *IBW* ideal body weight, *NA* not assessed, *TBW* total body weight, *BMI* body mass index, *BFM* body fat mass, *LBM* lean body mass, *RMR* resting metabolic rate, *DXA* dual-energy X-ray absorptiometry, *BIA* bioelectrical impedance analysis

^a^ indicates the percentage of patients adhering to the guideline regarding protein intake (≥60 g/day)

### Quality of individual studies

Four studies were free from a high risk of bias in all domains [25–28]. One study contained a high risk of bias based on funding [24]. In addition, in four of the five studies [24–27] the risk of bias was unclear concerning blinding. A summary of the risk of bias for the individual studies can be found in Table 2.

**Table 2:**
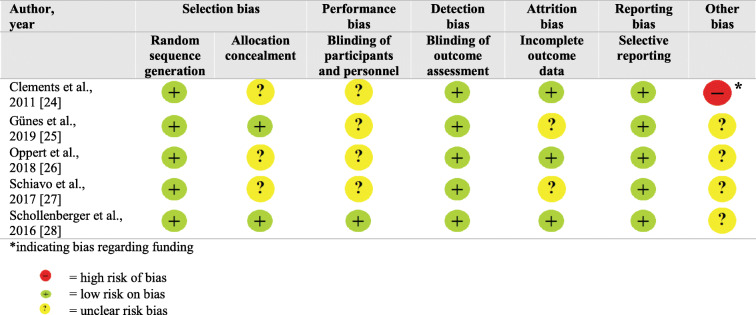
Assessment of risk of bias [24–28]

*indicating bias regarding funding

= high risk of bias

= low risk on bias

= unclear risk bias

### Lean body mass

All studies reported that LBM (kg) decreased significantly from pre-surgery to 8 weeks [24], 6 months [25, 26, 28] and 12 months [27] post-surgery. Two studies showed that protein supplementation [25] and a high-protein diet [27] resulted in significantly more preservation of LBM compared to control, respectively 8% vs. -12% and − 12% vs. -19% (Table 3). The other three studies demonstrated no differences towards LBM preservation following protein supplementation [24, 26, 28]. The studies that showed a significant difference in the decrease of LBM had an effect size of 0.31 [25] and 0.61 [27], which is considered small and moderate, respectively.

**Table 3:** Results of the individual studies

Author, year	LBM change (kg)(change%)	Effect size (d)	BFM change (kg)(change%)	TBW change (kg)(change%)	BMI change (kg/m^**2**^)(change%)	RMR change (kcal/day)
Clements et al., 2011 [24] ^1^	CON: − 7.9 ± 4.5 (14.6%)* PRO: − 7.7 ± 3.5 (14.7%)*	0.23 (− 0.48 to 0.95)	CON: − 8.0 ± 3.5 (14.3%)* PRO: − 9.2 ± 3.2 (15.7%)*	CON: −15.7 ± 2.5 (13.8%)* PRO: − 15.8 ± 2.6 (13.9%)*	CON: − 6.1 ± 1.1 (14%)* PRO: − 5.9 ± 0.9 (13.8%)*	CON: − 294.1 ± 207.2 (15.9%)* PRO: − 286.6 ± 271.1 (15.9%)*
Günes et al., 2019 [25] ^2^	CON: − 7.2 (12%)* PRO: 3.8 (8%)*^	0.31 (− 0.24 to 0.78)	CON: − 25.1 (41%)* PRO: − 36.7 (49.5%)*	CON: − 33.0 (26.9%)* PRO: − 33.1 (27.2%)*	CON: − 12.6 (27.4%)* PRO: − 12.2 (26.5%)*	*NA*
Oppert et al., 2018 [26] ^3^	CON: − 8.8 (− 10.1 to − 7.5)* (16%) PRO: − 8.2 (− 9.3 to − 7.1)* (15%)	*NA*	CON: −19.7 (− 21.5 to − 17.9)* PRO: − 19.8 (− 21.3 to − 18.2)*	CON: − 28 (− 30.6 to − 25.4)* PRO: − 27.2 (− 29.4 to − 25.1)*	CON:-10.5 (−11.4 to − 9.6)* PRO: − 10.2 (− 11.0 to − 9.4)*	*NA*
Schiavo et al., 2017 [27] ^1^	CON: − 14.5 (19%)* PRO: − 8.8 (12%)*^	0.61 (0.07 to 1.15)	CON: −23.7 (50%)* PRO: − 43.2 (84%)*^	CON: − 38.8 (31%)* PRO: − 46.7 (36%)*	*NA*	CON: − 645.16 (29%)*^ PRO: − 380.18 (17%)*^
Schollenberger et al., 2016 [28] ^1^	CON: − 7.8 (11.3%)* PRO: − 7.6 (11.7%)*	0.3 (− 0.59 to 1.18)	CON: −21.0 (30.8%)* PRO: − 29.1 (37.2%)*	CON: −28.7 (20.9%)* PRO: − 36.4 (25%)*	CON: −10.3 (21%)* PRO: − 13.0 (25%)*	*NA*

^1^ expressed as mean ± SD (change%)

^2^ expressed as mean (change%)

^3^ expressed as mean (CI) (change%)

*denotes significant difference from baseline. ^denotes significant difference from control

Abbreviations: *CON* control group, *PRO* protein group, *NA* not applicable or not assessed, *LBM* lean body mass, *BFM* body fat mass, *TBW* total body weight, *BMI* body mass index, *RMR* resting metabolic rate

### Total body weight, body fat mass, body mass index and resting metabolic rate

All studies reported that BMI [24–26, 28], TBW and BFM [24–28] decreased significantly following BS. None of the studies observed a significant difference in the decrease of TBW and BMI between the control and protein group. Only one study showed a significant difference in the reduction of BFM between the two groups, indicating a higher decrease in BFM following a high-protein diet [27]. Additionally, only one of the two studies that examined the effect of an additional protein intake on RMR demonstrated a significant higher RMR following a high-protein diet compared to a normal protein diet [27] (Table 3).

## Discussion

As result of bariatric surgery, patients are susceptible to protein deficiency which can result in an undesirable LBM loss. Evidence about the effect of protein supplementation or a high-protein diet (≥60 g/day) on LBM preservation is scarce. Therefore, this systematic review was conducted to evaluate these effects. Two of the five studies supported the hypothesis that protein supplementation or a high-protein diet resulted in significant LBM preservation [25, 27], whereas the other three studies did not support the hypothesis [24, 26, 28]. This discrepancy can be attributed to differences in protein intake, type of surgery and measurement tools which are discussed below.

The first explanation for why three studies failed to detect a significant LBM preservation is that the actual daily protein intake of these patients may have been too low. The studies that failed to demonstrate significant LBM preservation following protein supplementation reported an actual daily protein intake of 67 and 82 g/day [26, 28], though this amount is considered as adequate according to literature [6–8]. The actual daily protein intake in one of the studies that observed a significant LBM preservation was much higher, namely 143 g/day [27]. The other study that showed a significant LBM preservation reported a daily protein intake of just 51 g/day, while the protein intake of the control group was unknown [25]. The amount of 51 g/day should be criticized as protein intake was measured the first month after surgery and it is plausible that patients increased their protein intake hereafter, resulting in a higher protein intake at the time of measuring LBM (3 and 6 months). Based on the abovementioned findings (143 g/day resulting in significant LBM preservation [27]; 67 g/day to 82 g/day not resulting in LBS preservation [26, 28]), it could be questioned whether 60–80 g/day is sufficient to maintain LBM.

A lack of compliance might explain the relatively low actual protein intake within the first months after surgery. In the study of Oppert et al. this may have attributed to insignificant outcomes [26]. Unfortunately, none of the included studies reported clear causes for poor compliance. We speculate that this could be attributed to the occurrence of side effects, food intolerances and a lack of understanding regarding the need of adequate proteins [29, 30].

Protein intake and subsequent absorption may have been influenced by the type of surgery as both restrictive and malabsorptive procedures were included in this study. The two studies that found a significant effect included patients who underwent a restrictive procedure (SG) [25, 27], in contrast to the studies that included RYGB patients where no effect was found [24, 26, 28]. It is interesting to note that Schollenberger et al. reported, in a separate analysis of only SG patients, that protein supplementation led to significant LBM preservation [28]. An explanation for these findings may be that protein digestion and absorption is higher after restrictive surgery. This proposes that the additional protein intake is less effective in RYGB patients, but results in more pronounced LBM preservation in SG patients.

A third explanation may be the usage of different tools for measuring body composition (BIA versus DXA), both presenting important limitations. The two studies that detected a significant LBM preservation used BIA [25, 27]. However, BIA is known for overestimating LBM in bariatric patients and the validity of BIA is influenced by fatness [31–33]. As a result, the two significant outcomes are potentially more pronounced. Additionally, DXA is limited by the fact that the fat free mass compartment is measured rather than directly muscle mass [32]. Future research is recommended to measure body composition with a four-compartment model to overcome this limitation.

It is conceivable that physical activity might have influenced study outcomes as it is known from the field of sports physiology that physical activity plays an important role in LBM preservation [34–36]. Four of the five studies did not report anything about physical activity of the patients, implying uncertainty on whether and how physical activity influenced LBM preservation. The study that did report about physical activity (i.e., supervised strength training for 18 weeks plus additional protein intake), failed to show a significant preservation in LBM [26]. Contrarily, Muschitz et al. approved the synergistic effect of physical activity on protein supplementation as they observed significant LBM preservation [37]. The discrepancy in this outcome may be explained by the difference in study length, 18 weeks vs. 24 months respectively [26, 37]. Further studies investigating the synergistic effect of physical activity and protein supplementation in bariatric patients are limited, which implies that it is difficult to draw conclusions based on these two studies.

There are some methodological limitations in this systematic review which should be mentioned. Four of the five studies lacked (double) blinding, which could have influenced the study outcomes. Furthermore, only two of the five studies reported high compliance to protein intake and because of this, outcomes are potentially less pronounced than expected. Moreover, the number of the included studies in this systematic review is small, potentially resulting in a relative low power of this systematic review. A further comparison of the included studies was complicated due to heterogeneity of the study protocols (e.g. supplementation type, dose and timing) and the measurement tools.

New studies investigating the most effective dose of supplements to preserve LBM in post-bariatric surgery patients are warranted as perhaps the dose of 60–80 g/day is insufficient to maintain muscle mass. In addition, it is advised to conduct studies examining the most effective composition of protein supplements (e.g. whey vs. casein vs. soy) in order to enable interstudy comparison. Special attention needs to be paid to the effect of leucine on LBM preservation, given its key role in muscle protein synthesis. On top of that, studies focusing on the synergistic effect of physical activity and protein intake on LBM preservation are warranted.

## Conclusion

Although the preservation of LBM in post-bariatric surgery patients is of extreme importance, our systematic review resulted in the inclusion of only five studies. These studies showed inconclusive evidence for LBM preservation due to protein supplementation or a high-protein diet. Notwithstanding, this work offers awareness to current healthcare providers who should prompt an adequate protein intake in post-bariatric surgery patients. More specific and personalized recommendations regarding protein intake may need to be established by high quality research. New studies investigating the quantity (g/day) and quality (whey, casein or soy) of protein supplements or high-protein diets, possibly in combination with resistance training, in larger study populations are needed.

## Supplementary Information

**Additional file 1.** Appendix Table 1. Search strategy MEDLINE, EMBASE and Cochrane.

## Data Availability

The datasets used and/or analysed during the current study are available from the corresponding author on reasonable request.

## Abbreviations

BIA: Bioelectrical impedance analysis
BFM: Body fat mass
BMI: Body mass index
BS: Bariatric surgery
DXA: Dual-energy X-ray absorptiometry
IBW: Ideal body weight
LBM: Lean body mass
MRI: Magnetic resonance imaging
RCT: Randomized controlled trial
RMR: Resting metabolic rate
SG: Sleeve gastrectomy
RYGB: Roux-en-Y gastric bypass
TBW: Total body water
