# Supplementary material for: The effect of additional protein on lean body mass preservation in post-bariatric surgery patients: a systematic review
Source: Nutr J. 2021 Mar 14;20:27. doi: 10.1186/s12937-021-00688-3 (PMC7958440; doi:10.1186/s12937-021-00688-3)
Supplement: Supplementary file 1 — Additional file 1. Appendix Table 1. Search strategy MEDLINE, EMBASE and Cochrane. [file 12937_2021_688_MOESM1_ESM.docx]

**Table 1.** Search strategy MEDLINE, EMBASE and Cochrane

**Search strategy MEDLINE (PubMed):**

| #1 | "Bariatric Surgery"[Mesh] OR bariatric surger*[tiab] OR bariatric surgical[tiab]OR metabolic surger*[tiab] OR stomach stapling[tiab] |
| --- | --- |
| #2 | gastric bypass*[tiab] OR gastroileal bypass*[tiab] OR gastrojejunostom*[tiab] |
| #3 | obesity surger*[tiab] |
| #4 | #1 OR #2 OR #3 |
| #5 | "Dietary Proteins/administration and dosage"[Mesh] |
| #6 | protein* AND supplement*[tiab] |
| #7 | amino acid supplement*[tiab] |
| #8 | #5 OR #6 OR #7 |
| #9 | #4 AND #8 |

**Search strategy EMBASE (Ovid)**

| 1. | exp bariatric surgery/ |
| --- | --- |
| 2. | (bariatric surger* or bariatric surgical or metabolic surger* or stomach stapling).ab,kw,ti. |
| 3. | (gastric bypass* or gastroileal bypass* or gastrojejunostom*).ab,kw,ti. |
| 4. | "obesity surger*".ab,kw,ti. |
| 5. | 1 or 2 or 3 or 4 |
| 6. | exp protein intake/ |
| 7. | (protein* and supplement*).ab,kw,ti. |
| 8. | "amino acid supplement* ".ab,kw,ti. |
| 9. | 6 or 7 or 8 |
| 10. | 5 and 9 |

**Search strategy Cochrane Library**

| #1 | (bariatric surger*):ti,ab,kw OR ("bariatric surgical"):ti,ab,kw OR (metabolic surger*):ti,ab,kw OR ("stomach stapling"):ti,ab,kw OR (gastric bypass*):ti,ab,kw |
| --- | --- |
| #2 | (gastric bypass*):ti,ab,kw OR (gastroileal bypass*):ti,ab,kw OR (gastrojejunostom*):ti,ab,kw OR (obesity surger*):ti,ab,kw |
| #3 | #1 OR #2 |
| #4 | (protein*):ti,ab,kw OR ("amino acid" or "amino acids"):ti,ab,kw |
| #5 | (supplement*):ti,ab,kw |
| #6 | #4 AND #5 |
| #7 | #3 AND #6 |
